# Design and Evaluation of a New Type of Knee Orthosis to Align the Mediolateral Angle of the Knee Joint with Osteoarthritis

**DOI:** 10.1155/2012/104927

**Published:** 2012-04-19

**Authors:** Amir Esrafilian, Mohammad Taghi Karimi, Arezoo Eshraghi

**Affiliations:** ^1^Mechanical Engineering Department, Isfahan University of Technology, Isfahan, Iran; ^2^Orthotics and Prosthetics Department, Rehabilitation Faculty, Isfahan University of Medical Sciences, P.O. Box 81745-164, Isfahan, Iran; ^3^Biomechanical Engineering Department, Malaya University, Kuala Lumpur, Malaysia

## Abstract

*Background*. Osteoarthritis (OA) is a disease which influences the performance of the knee joint. Moreover, the force and moments applied on the joint increase in contrast to normal subjects. Various types of knee orthoses have been designed to solve the mentioned problems. However, there are other problems in terms of distal migration during walking and the alignment of the orthosis which cannot be changed following the use of brace. Therefore, the main aim of the research was to design an orthosis to solve the aforementioned problems. *Method*. A new type of knee orthosis was designed with a modular structure. Two patients with knee OA participated in this research project. The force applied on the foot, moment transmitted through the knee joint, and spatiotemporal gait parameters were measured by use of a motion analysis system. *Results*. The results of the research showed that the adduction moment applied on the knee joint decreased while subjects walked with the new knee orthosis (*P*-value < 0.05). *Conclusion*. The new design of the knee brace can be used as an effective treatment to decrease the loads applied on the knee joint and to improve the alignment whilst walking.

## 1. Introduction

Osteoarthritis (OA) is a disease which influences the body joints. In this disease the articular surface of the joint is damaged and the smooth motion of the joint is disturbed. It is the most common type of arthritis which affects more than 13% of the American people aged 55–64 and more than 70% with the age of 65–74. By the year 2020, an estimated 18.2% of the population will be affected in the United States of America [1, 2]. Patients with knee OA usually demonstrate major involvement in medial compartment. The most common types of alteration include decreasing knee joint excursion, altered ground reaction force applied on the leg, and altered muscle pattern of key lower extremity muscles involved in gait [2].

Force augmentation may be a contributing factor in the development of knee OA. The force applied on the knee is not transmitted equally between the medial and lateral compartments while walking. The loads applied on the medial compartment are 2.5 times more than the loads on the lateral side. In healthy subjects, between 71% and 91% of total knee force is transmitted through tibiofemoral compartment compared to 100% in OA [3]. There are various types of treatment to manage knee varus deformity associated with OA, to align the mechanical axis of the limb, which include wedge osteotomy of the tibia, using various kinds of knee brace, lateral wedge insoles, and subtalar strap [4–11]. However, most clinicians attempt to use conservative treatment instead of operative methods [5, 6, 12, 13].

The two most common types of conservative treatment for patients with OA are various kinds of knee braces and lateral wedge insole, which have been used to reduce knee pain, to improve knee alignment, and to increase the knee joint range of motion during walking [4, 5, 7–10, 14–16]. Using lateral wedge insole is another conservative treatment used in this regard. Sasaki and Yasuda (1987) showed that lateral wedge alters the mechanical alignment of the lower limb and reduces the loads applied on the medial compartment of the knee [17]. On the contrary, it was defined from the results of the double-blind randomized crossover trial in 90 patients with knee OA that the effect of lateral wedge insole was neither statistically significant nor clinically important [18]. Knee brace has been proved to have some benefits for the patients, such as reducing the knee joint varus moments, improving the configuration of the loads applied on the knee, and decreasing pain during walking [19, 20]. Valgus braces attempt to reduce excessive compartmental loading and improve the performance of the subjects. The braces unload the painful compartment by applying a three-point pressure system. Various kinds of knee braces, such as Generation II brace, medial unloading Monarch brace, and Vista CA brace have been used for patients with knee OA [19–23]. However, patients experience some problems with use of knee orthoses such as tendency of the brace to migrate distally as a result of muscles contraction [6, 19–22]. Moreover, it is not possible to change the alignment of the brace during follow-up period. Therefore, the aim of this project was to design and evaluate an orthosis to overcome the aforementioned problems. The main hypotheses associated with this research study were the orthosis improves the performance of the subjects while walking and also decreases the mediolateral instability of the knee joint.

## 2. Method

A new type of knee orthosis has been designed which has a modular structure to change the alignment based on the patients' need. The components of the orthosis were designed so that they can be aligned with respect to each other. It allows alignment adjustment while the user wears the orthosis. The orthosis was made of three main components which include the upper shell, the lower shell, and the knee joint.

**Figure 1:**
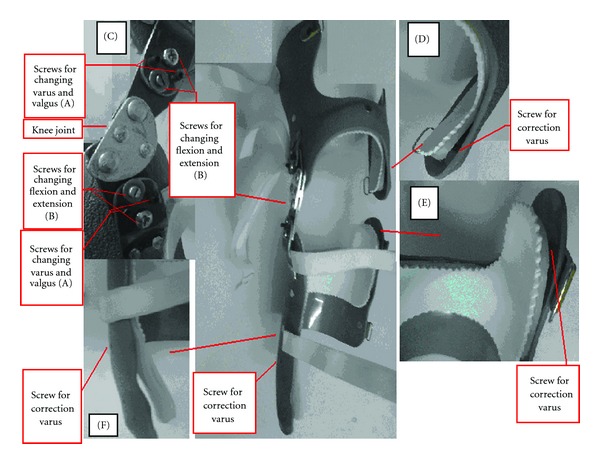
The new design of the knee orthosis designed for patients with knee OA. C: the knee joint. D: the lower medial part of thigh shell. E: the upper lateral part of shank shell with the aligning screw. F: the lower medial part of the orthosis with the aligning screw.

The upper shell of the orthosis was made of polyethylene (high density) vacuumed on the positive cast of the patients' limb. It had two elastic straps which secured the orthosis on the leg (Figure 1). The upper shell of the orthosis worked as the third component of three-point pressure system. The lower shell of the orthosis consisted of two layers. The first layer was made of low-density polyethylene which was vacuumed over the positive cast. It was trimmed to cover the upper lateral and the lower medial parts of the leg. These areas were the locations of corrective forces used to align the limb. The second layer, which was made of high-density polyethylene, was vacuumed on the first layer. The first layer could be moved inside the second one by aligning screws in the upper lateral and lower medial sides of the leg. Therefore, the magnitude of the force could be adjusted according to the patient's need. The lower shell was secured on the leg by three elastic straps, Figure 1.

The knee joint of the orthosis was a polycentric type with special components to align the orthosis in the anteroposterior and mediolateral directions. The screw A in Figure 1 allowed orthotists to change abduction/adduction alignment of the orthosis. The screws B helped orthotists to change the alignment of the orthosis in the sagittal plane.

**Subjects**

Two patients with knee OA without any other reported musculoskeletal disorders participated in this research project. The mean values of their mass, height, and age were 59 kg, 1.6 meter, and 53 year, respectively. Subjects were diagnosed with medial compartment knee OA according to the American College of Rheumatology criteria for the diagnose of knee OA, medial knee pain, and radiographic osteophyte at the medial joint space of the knee [24]. The severity of knee OA was defined by the use of the Kellgren and Lawrence grade (K-L grade) based on the X-ray of the knee, as it was described in the Atlas of Standard Radiography [25]. The subjects were asked to walk on a level surface with and without orthosis to investigate the influence of orthosis on the knee alignment. Ethics approval was received from Isfahan University Ethics Committee before starting the data collection. The subjects were asked to sign a consent form.

**Parameters**

Some spatiotemporal gait parameters, knee joint angle in sagittal and frontal planes, the mediolateral moment applied to the knee joint, vertical and mediolateral forces transmitted through the leg, and the hip joint flexion/extension angles were evaluated during walking with and without the knee orthosis.

**Procedure**

For tracing the movement of the subjects, an array of 7 high-speed cameras by Qualysis Company was used. Moreover, the force applied on the leg was measured by a Kistler force plate. The analyse space for cameras was calibrated by moving and rotating a rod with reflective markers in space. The motions of the markers and the force plate data were recorded by use of Track Manager Software produced by Qualysis Company. The markers were labelled and defined in Track Manager and export as 3D files. The subjects lower body anatomy was reconstructed by Visual 3D software produced by C Motion Company. This programme was also used for calculation of angle change of the hip, knee, and ankle joints during walking. Force plate data was also processed with Visual 3D to calculate resulting moments of the lower limb joints.

The marker set based on six degrees of freedom principles, primarily using marker cluster for tracing, based on CAST/ISB recommendations [26]. The markers used in this research project had 14 mm diameter and were attached to the body according to the preferred method of marker fixation which has been used in the Bioengineering Unit of Strathclyde University. Sixteen markers were attached to the right and left anterior superior iliac spines (ASIS), right and left posterior superior iliac spines (PSIS), right and left medial and lateral malleolus, right and left medial and lateral sides of the knee joints, and first and fifth metatarsal heads. Knee markers were attached on the skin of the medial and lateral sides of the knee joint while the subjects wore the orthosis. Moreover, four marker clusters comprising of four markers attached on the rhomboid plates were attached to the anterolateral surfaces of the legs and thighs by use of extensible Velcro straps. The subjects were trained to maintain an even gait with the orthosis for one hour before data collection. The subjects were asked to walk along a level surface to collect five successful trials (the subjects were asked to repeat the trials separately). The collected data were filtered (Woltring filter with frequency of 10 Hz) and split to gait cycle interval using heel strike data.

The parametric statistical test was used to evaluate the difference between the mean values. The two sample *t*-test with a significance level of 0.05 was used for the final analysis.

## 3. Results

The mean values of the gait parameters are shown in Table 1. As can be seen, the mean values of the moments applied on the knee joint during walking with orthosis in both subjects were significantly less than that of normal walking (*P*-value < 0.05). The instability of the knee joint, which was represented as the excursion of the knee joint motion in the frontal plane, also decreased significantly during walking with orthosis in contrast to that in normal walking. The values were 21.43 ± 2.65 and 15.39 ± 4.1 degree in the first subject while walking without and with the knee orthosis, respectively (*P*-value = 0.001). The mediolateral force applied on the limb decreased in walking with orthosis (the difference of the mean values was significant; (*P*-value < 0.05)).

**Figure 2:**
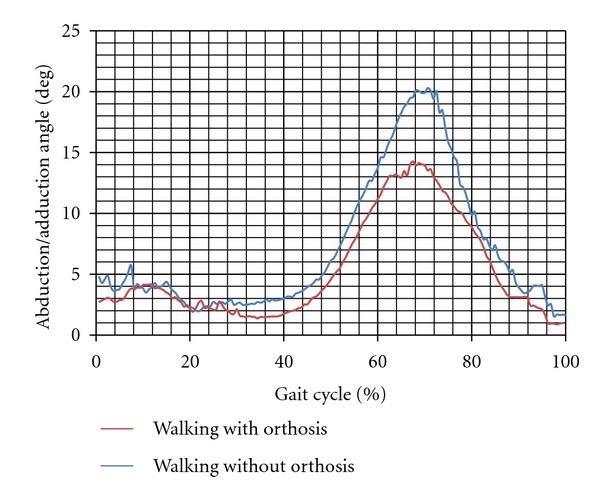
Abduction/adduction angle of the knee joint while walking with and without orthosis (subject 1).

**Figure 3:**
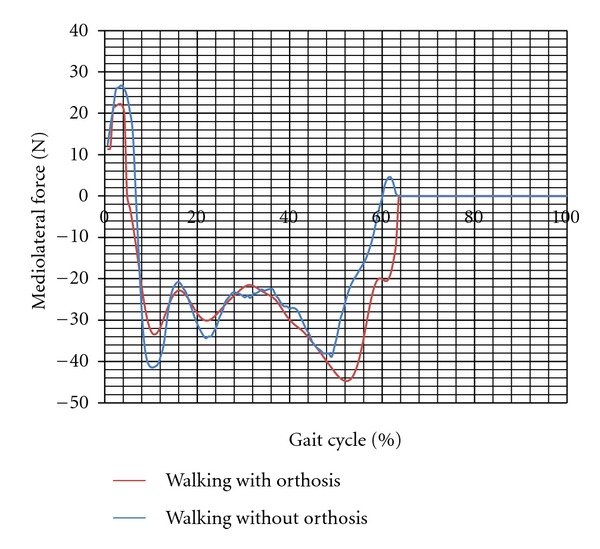
Mediolateral force applied on the limb while walking with and without orthosis (subject 1).

**Figure 4:**
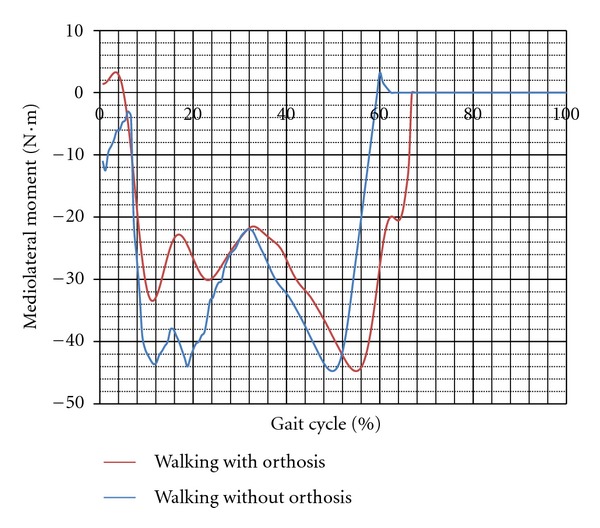
Mediolateral moment applied on the knee joint while walking with and without orthosis (subject 1).

**Figure 5:**
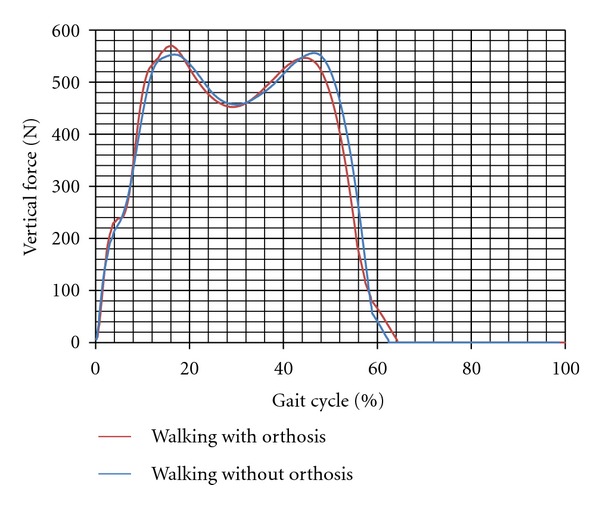
The vertical force applied on the foot while walking with and without knee orthosis (subject 1).

There was no significant difference between the vertical force applied on the leg in the first subject while walking with and without the orthosis (573 ± 36 compared to  560.9 ± 29 N). In contrast in the subject 2, the vertical force transmitted through the leg with and without the brace was  688 ± 17  and  642 ± 12  N, respectively (*P*-value = 0.001). Moreover, the mediolateral force decreased while walking with the orthosis compared to without orthosis (*P*-value < 0.05). Figures 2, 3, 4, and 5 show the moment applied on the knee, vertical and mediolateral forces applied on the limb, and adduction of the knee joint during walking with and without the orthosis.

## 4. Discussion

It is somewhat controversial whether using knee orthosis reduces the mediolateral moment applied to the knee joint or not [5]. It is expressed by some researchers that the main influence of unloader brace in most cases is compensation for a portion of the external load [5]. However, the results of this research showed that the adduction moment applied to the knee joint decreased significantly following the use of a new design of knee brace. It is clear that the two important parameters which influence the moment applied on the knee are the magnitude of the mediolateral force and its moment arm. As can be seen from Table 1, the mediolateral force decreased following use of the knee orthosis. Moreover, improvement of the knee joint alignment in the mediolateral direction is the other important finding of this research.

The mediolateral force applied to the knee joint decreased significantly (*P* value was less than 0.05) with the use of the knee brace in the current research. However, the results of the research undertaken by Schmalz et al. (2011) showed that using the knee orthosis did not influence the adduction force applied to the foot [21]. In contrast, in the current research, there was an increase in the magnitude of the vertical force, especially in subject 2. Reduction in the valgus angle of the knee joint was interpreted as the main reason for the decrease in the mediolateral loads transmitted through the medial compartment of the knee.

Most studies reported only a few degrees of varus angle reduction during gait [5]. The results of the current research study also showed that the valgus angle of the knee joint decreased, particularly during the last part of the stance phase. Therefore, it might be concluded that even though the angular change was small, it seems reasonable that the valgus angle reduction would lead to the decreases in the loads transmitted through the knee joint in the mediolateral plane. To the authors' knowledge, the tibiofemoral angle has been measured in some research studies by use of X-ray in a static quiet standing position. However, we measured the angle of the knee joint in the mediolateral direction during walking, which might be a good alternative method in this regard.

The magnitude of orthosis distal migration was not directly measured in this study. It is clear that the congruency of the anatomical and mechanical knee joints influences the performance of the orthosis. If the orthosis migrates distally during walking, it will decrease the knee joint range of motion in the sagittal plane, and the patient will have problems during walking. However, in the current research, the range of motion of the knee joint was nearly the same while walking with and without orthosis. Therefore, it might be indirectly concluded that the distal migration of our new orthosis was not too much to influence the performance of the subject.

The new design of the orthosis might have the following advantages over the other designs.

- It has a modular structure to allow changing the alignment of the components with respect to each other.
- The distal migration of the orthosis is not too much to influence the function of the orthosis.
- The magnitude of the corrective force can be changed according to the patient's needs.

The followings are some limitations of this research study which need to be acknowledged.

- The number of subjects was too limited. It is recommended to evaluate the orthosis on more OA patients.
- The quality of life of the subjects following the use of orthosis was not evaluated.
- The knee joint pain severity was not evaluated.

Finally, it is recommended that other parameters such as stability of the subjects, energy consumption during walking, and severity of the knee pain be measured in future studies.

## 5. Conclusion

The mediolateral force transmitted through the knee joint was decreased following the use of a new design of knee brace. Moreover, the moment applied to the knee joint declined which influenced the pain associated with Knee OA. Distal brace migration on the leg is often a compliant of the patients, especially during extension. It was solved in the current designed orthosis by use of the elastic straps.

##  Disclosure

The results of this research can be used by most of the clinicians regarding treatment of patients with Knee OA. A new design of orthosis has been introduced in this paper which can be used for most patients with knee OA.

## Figures and Tables

**Table 1:** The values (mean ± SD) of the gait parameters during walking with and without knee orthosis.

	Parameter	Flexion angle (knee)	Adduction angle (knee)	Flex/Ext angle (hip)	^*¹*^Force 1 (N)	^²^Force 2 (N)	^³^Moment (Nm)
Subject 1	Without orthosis	64.86 ± 5.7	21.43 ± 2.65	43.6 ± 2.07	560.9 ± 29	51.4 ± 12.5	78.56 ± 32.43
	With orthosis	54.35 ± 1.02	15.39 ± 0.41	44.2 ± 0.96	573 ± 36	43.06 ± 12.1	54.1 ± 12
	*P*-value	0.004	0.001	0.056	0.56	0.031	0.52

Subject 2	Without orthosis	66.52 ± 5.6	5.06 ± 1.6	41.6 ± 3.2	642.5 ± 12	46.2 ± 4.2	87.36 ± 36.7
	With orthosis	60.66 ± 1.87	4.5 ± 0.37	40.6 ± 2.6	688 ± 17	40.45 ± 3.4	66.5 ± 25.6
	*P* value	0.049	0.11	0.5	0.001	0.044	0.525

^1^Vertical force.

^2^Mediolateral force.

^3^Mediolateral (adductor) moment.
